# The impact of health vs. non-health goals on individuals’ lifestyle program choices: a discrete choice experiment approach

**DOI:** 10.1186/s12889-020-8416-3

**Published:** 2020-03-30

**Authors:** Tim M. Benning, Benedict G. C. Dellaert, Theo A. Arentze

**Affiliations:** 1 Department of Applied Economics, Erasmus School of Economics, Erasmus University Rotterdam, Rotterdam, The Netherlands; 2 Department of Business Economics, Erasmus School of Economics, Erasmus University Rotterdam, Rotterdam, The Netherlands; 3 Monash Business School, Monash University, Melbourne, Australia; 4 Urban systems and real estate, Department of the Built Environment, Eindhoven University of Technology, Eindhoven, The Netherlands

**Keywords:** Discrete choice experiment, Goals, Lifestyle program, Preferences

## Abstract

**Background:**

Goals play an important role in the choices that individuals make. Yet, there is no clear approach of how to incorporate goals in discrete choice experiments. In this paper, we present such an approach and illustrate it in the context of lifestyle programs. Furthermore, we investigate how non-health vs. health goals affect individuals’ choices via non-goal attributes.

**Methods:**

We used an unlabeled discrete choice experiment about lifestyle programs based on two experimental conditions in which either a non-health goal (i.e., looking better) or a health goal (i.e., increasing life expectancy) was presented to respondents as a fixed attribute level for the goal attribute. Respondents were randomly distributed over the experimental conditions. Eventually, we used data from 407 Dutch adults who reported to be overweight (*n* = 212 for the non-health goal, and *n* = 195 for the health goal).

**Results:**

Random parameter logit model estimates show that the type of goal significantly (*p* < 0.05) moderates the effect that the attribute diet has on lifestyle program choice, but that this is not the case for the attributes exercise per week and expected weight loss.

**Conclusions:**

A flexible diet is more important for individuals with a non-health goal than for individuals with a health goal. Therefore, we advise policy makers to use information on goal interactions for developing new policies and communication strategies to target population segments that have different goals. Furthermore, we recommend researchers to consider the impact of goals when designing discrete choice experiments.

## Background

Individuals’ goals play an important role in the choices that they make [1–4]. The traditional psychology literature specifically emphasizes the importance of goals, and goal pursuit, for explaining individuals’ choices [5–7]. Goals also play a key role in individuals’ health related decisions. For example, Segar et al. [8] investigated the participation in physical activity between healthy midlife women with distinct goals and found that women with weight loss and health benefit goals participated in less physical activity than those with sense of well-being and stress reduction goals. Another particularly clear example is individuals’ choices for lifestyle programs, as this is a context where it is very common for individuals to set explicit goals. For example, a participant of a lifestyle program who wants to lose weight to look better may choose a different program (i.e., attaches different values to specific program attributes) than a participant who wants to lose weight to increase life expectancy.

A widely used method to analyze individuals’ decisions in the field of health care are discrete choice experiments (DCEs) [9, 10]. In a DCE, individuals complete several choice tasks by choosing one of the offered alternatives – which have (different) attributes with varying attribute levels [11, 12]. These attributes typically represent key characteristics of the alternatives [11, 12]. The resulting models of individuals’ decisions then directly connect changes in these characteristics on the probability that a certain alternative will be selected.

In the literature that measures individuals’ preferences of lifestyle programs by means of discrete choice experiments [13–24], several studies included attributes related to the expected outcome (usually weight loss) of the lifestyle program [16, 19–21, 24]. More importantly, other studies took into account goal-related information by including a goal-attribute in the DCE [14, 22]. For example, Johnson et al. [14] used an attribute called ‘weight loss goal in a year’ with the attribute levels ‘no weight loss’, ‘lose 20 lb’, and ‘lose 40 lb’. Furthermore, Ryan et al. [22] included an attribute named ‘short-term goal’ and the levels ‘look better’, ‘feel better’, and ‘look better and feel better’. Both studies analyzed the effect of a goal-related attribute on individuals’ lifestyle program choices [14, 22]. However, to the best of our knowledge, the question how including goals in a DCE affects individuals’ choices via (a moderating effect on) other non-goal attributes has not been answered yet. An answer to this question would be a relevant contribution to the literature, because the goal itself does not only affect the choices that individuals make, but it also matters – and this may be even more important – how the goal individuals have in mind affects the importance of other non-goal related characteristics of the programs individuals choose between.

We predict that including and experimentally manipulating goals in a DCE significantly shifts the importance of the attributes related to the attainment of that goal. The reason for this prediction is that individuals who pursue a specific goal in a decision task are likely more conscious of the goal, which enables them to use conscious strategies to identify attributes that are specifically relevant for the goal. For most individuals, this promotes choosing the alternative that is most consistent with the goal [25]. Because of the fact that the attention on incorporating broader outcome measures into economic evaluations is increasing [26–30], we specifically investigate the effect of presenting non-health versus health goals. This distinction between non-health and health goals also plays an important role in the case of lifestyle programs [22]. Our study is not only relevant in a health promotion context, but also for the broad literature on DCEs in health care. If researchers would take into account the (moderating) effect that goals have on the relative importance of other non-goal attributes in DCEs, this could lead to more precise utility estimates and therefore better predictions – given that individuals’ potential future benefits are now taken into account [31].

## Methods

### Discrete choice experiment

In this section, we propose the set-up of a DCE that includes goals and develop it for lifestyle program choices – a context in which individuals commonly set and pursue goals.

### Attributes and attribute levels

We performed a literature search (July 2018) and found 12 articles in which a DCE was used to measure individuals’ preferences for lifestyle programs [13–24]. The attributes were selected based on their frequency of use in the aforementioned literature and their relevance for our study. In the literature, attributes related to exercising, costs, and diet were most often used. Because of the fact that the costs of lifestyle programs for individuals with overweight-related health risks will be reimbursed (basic insurance) in the Netherlands from 2019 onwards, we decided to include the attributes ‘exercise per week’ [14] and ‘diet’ [14, 23], but no cost attribute. Furthermore, we included the attributes ‘expected weight loss’ and ‘goal’ so that the combination of all attributes in the choice tasks resembles individuals’ goal-related reasoning and decision making in real life. This meant that the choice tasks were created in such a way that a respondent could reason as follows: “If I exercise (… hours) per week and follow a (… diet) I will likely lose (… kg) of weight in 6 months, this helps me to reach my goal of (…). Therefore, I choose program (…)”. Of course, the main reason why we included the goal attribute was to determine how different goals affect the relative importance of non-goal attributes. To reduce cognitive burden of respondents we included only four attributes in our DCE [23].

For the attribute ‘exercise per week’, we used 2, 4, and 6 h as attribute levels as in Alayli-Goebbels et al. [18]. For the attribute ‘diet’, we used the attribute levels ‘flexible low-calorie diet’ and ‘restricted diet’ as in Johnson et al. [14] and Chen et al. [23]. Note that we excluded the lowest attribute levels of the aforementioned attributes (i.e., ‘0 h’ and ‘no diet’) since only exercising or only following a diet would, according to current Dutch health insurer policies, not lead to full reimbursement by basic insurance. This policy dictates that full reimbursement of lifestyle programs by health insurance coverage in the Netherlands is only received if lifestyle programs focus on improvements in both healthy behavior as well as food consumption habits (https://www.zorgwijzer.nl/zorgverzekering-2019/hulp-bij-overgewicht-gedekt-met-basisverzekering). Furthermore, we used the attribute levels 5 kg and 10 kg for the expected weight loss attribute as in Veldwijk et al. [19], Wanders et al. [21], and Salempessy et al. [24], but for similar reasons excluded the 0 kg attribute level. By doing so, the attribute level range of the weight loss attribute aligned with the range of the exercise and diet attributes.

### Goals

The psychology literature on goals distinguishes between goal-setting and goal-striving [32–34]. Goal-setting eventually leads to goal intentions, which can be behavioral intentions (something that an individual intends to do) and end-state intentions (something an individual intends to achieve) [34]. These end-state intentions are most in line with how we define goals in this study, given that we mainly focus on the specific outcome that an individual achieves – and the fact that the attributes (i.e., dieting and exercising) generally represent means towards this outcome. In line with this focus on end-states, we follow the definition of goals as cognitive representations of desired (end) states [5, 6].

There is an increasing attention in the economic welfare literature for incorporating broader aspects of consumer evaluations such as happiness and wellbeing into economic evaluations [29, 30]. Similar broader perspectives are also being investigated in public health economics [26–28]. In the context of lifestyle programs, non-health aspects of interventions can also be relevant for individuals, as they may choose to participate in programs for other reasons than health alone – which is closely related to the goal-based decisions that we investigate in the present study [35]. Because of the increasing attention for incorporating broader outcomes, we specifically distinguish between non-health and health outcomes. More specifically, we used the (positively framed) outcomes (i.e., end-states) of ‘looking better’ and ‘increasing life expectancy’ to operationalize respectively the non-health and health goal. These goals are relevant and common for individuals participating in a lifestyle program [8, 36].

### Experimental design

We created an unlabeled D-efficient design for the multinomial logit (MNL) model based on two alternatives and 16 choice tasks without an opt-out option in Ngene 1.2 (Choice Metrics 2018). This design was used for both the non-health goal as well as the health goal condition. In a specific goal condition, the goal attribute contained a fixed attribute level in the choice tasks and only differed between conditions. This means that we used the same design for each goal, and treated goal as a kind of context variable [37]. The reason why we propose a design in which a fixed attribute level of the goal attribute was presented in all choice tasks of a specific goal condition, was that we tried to resemble individuals’ real life decision making based on goals as close as possible. In recent literature on goals, we found that individuals use one or more goals as input for decision-making [4], but it is highly likely that they do not use new goals for repeating decisions of similar nature – as is the case with the fixed goal in the choice tasks of our DCE. For an overview of the attributes, attribute levels and experimental conditions used, we refer to Table 1.

**Table 1:** Attributes and levels of the lifestyle programs in the DCE for each goal condition

Attributes	Attribute levels	
	Non-health goal condition	Health goal condition
Diet	Flexible low-calorie diet	Flexible low-calorie diet
	Restricted diet	Restricted diet
Exercise per week	2 h of exercise	2 h of exercise
	4 h of exercise	4 h of exercise
	6 h of exercise	6 h of exercise
Expected weight loss	5 kg	5 kg
	10 kg	10 kg
Goal ^a^	Looking better by losing weight	Increasing life expectancy by losing weight

DCE indicates discrete choice experiment

^a^ The goal attribute had a fixed attribute level in each goal condition

Based on a pilot study (*n* = 58) prior parameters were obtained for the generation of the final experimental design. Within choice task alternative repetition, strict attribute level dominance, and choice task repetition were prevented in the design. Furthermore, we used the mfederov algorithm and focused on the mean value when computing the D-error. Eventually, thousand Halton draws were used in the design generation process.

The model for which the design was optimized included dummy coded variables for diet (two levels), exercise (three levels), and weight loss (two levels), but no constant. We took into account the prior parameter estimates and standard errors derived from the aforementioned pilot test. Interactions between the diet and weight loss, and the exercise and weight loss dummy variables were also included (based on zero priors). The final design had the same specification as the design for the pilot study, except that the aforementioned parameter priors for (the dummy variables of) the attributes were included to generate a more optimal design.

### Survey and data collection

We used two different online survey versions (see Additional file 1) that were created in Qualtrics. The survey versions differed from each other based on the two experimental conditions for the goal attribute (i.e., a non-health vs. health goal).^1^ This made it possible to investigate if using different goals would shift the importance of the attributes in the DCE. The survey versions were randomly distributed among respondents to obtain a close to equal number of completes for each goal condition, and prevent selection bias.

The survey started with questions about respondents’ age, gender, weight and height. Based on these questions, their body mass index (BMI) was calculated and presented to them. Next, respondents were asked to indicate whether their BMI was higher (or equal to) or lower than 25. Only respondents who reported a BMI higher than (or equal to) 25 could continue the survey. Then, respondents were informed about the usefulness of lifestyle programs for losing weight and our interest in understanding their preferences for different lifestyle programs. This was followed by an explanation of the attributes and attribute levels of the lifestyle programs and a scenario in which the general practitioner recommended respondents to participate in a specific fully reimbursed lifestyle program of 6 months. After this general scenario, respondents were asked to complete one example choice task (which was not used in the analysis) and 16 main choice tasks. In all choice tasks, they had to imagine that they were highly interested in participating in the type of lifestyle programs presented to them and, subsequently, they were asked to select their preferred lifestyle program out of two presented programs in each choice task. The lifestyle programs only differed on the attributes diet, exercise per week, and expected weight loss given that the attribute level of the goal attribute was constant for all choice tasks in each condition. In Table 2, an example of a choice task for the non-health goal condition is shown.

**Table 2:** Example of a choice task for the non-health goal condition

Features	Program A	Program B
Diet	restricted diet	flexible low-calorie diet
Exercise per week	6 h of exercise	2 h of exercise
Expected weight loss	10 kg	5 kg
Goal	looking better by losing weight	looking better by losing weight
I prefer:	Program A □	Program B □

After they had answered all choice tasks, respondents were asked how important several pre-selected goals (e.g., losing weight, feeling fitter, reducing risk of overweight related diseases, looking better, increasing life expectancy, and improving endurance) were for them on a 5 point Likert scale ranging from very unimportant to very important, and how healthy they normally feel (on a 0–100 scale). Furthermore, they were asked about the highest education level they obtained, whether they currently follow a diet, the average number of hours they exercise per week, their expected ability to finish a lifestyle program that lasts 6 months, and their intention to participate in a lifestyle program in the next 5 years.

Data was collected via a Dutch survey-sampling provider in the period mid-October to the end of October 2018. On an online platform, members of the survey provider’s Dutch propriety panel received a link to the online survey. After completing the survey, respondents were incentivized in the form of ‘panel points’, which could be exchanged for money, gift cards, or vouchers. The survey was pilot tested by the survey-sampling provider before the full launch.

### Econometric model

We analyzed the data using a random parameter logit (RPL) model in Nlogit 6.0 (Econometric Software, Inc.) based on 500 Halton draws for which start values were obtained from a MNL model. The utility function of the RPL model is specified below:

$$ {U}_{njt}=\alpha +\left({\beta}_1+{v}_{1n}\right)\ast EXERCIS{E}_j+\left({\beta}_2+{v}_{2n}\right)\ast DIET\_{FLEX}_j+\left({\beta}_3+{v}_{3n}\right)\ast WEIGHTLOSS\_{5}_j+\left({\beta}_4\right)\ast NONHEALTHGOAL\_ EXERCIS{E}_j+\left({\beta}_5\right)\ast NONHEALTHGOAL\_ DIE{T}_j+\left({\beta}_6\right)\ast NONHEALTHGOAL\_W{EIGHTLOSS}_j+{\varepsilon}_{njt} $$

In this utility function, *U*_*njt*_ is the utility of individual *n* related to a specific lifestyle program *j* in choice observation *t*. Here, the constant *α* is included to allow for a possible tendency (bias) to choose the first presented alternative in each set (all else equal). *β*_1_ to *β*_3_ are coefficients for the variables *EXERCISE*, *DIET* _ *FLEX* and *WEIGHTLOSS* _ 5 in program *j*. *v*_1*n*_ to *v*_3*n*_ are individual-specific error terms related to possible heterogeneity in the preference parameters *β*_1_ to *β*_3_. *EXERCISE* represents the number of hours of exercise in a week and is included as a continuous variable. *DIET* _ *FLEX* is a dummy variable for a flexible versus restricted diet, coded 1 if the diet is a flexible diet and 0 (i.e., base level) in case of a restricted diet. *WEIGHTLOSS* _ 5 is a dummy variable that indicates the expected weight loss after participating in the lifestyle program, coded 1 if the expected weight loss resulting from participating in the lifestyle program is 5 kg and 0 (i.e., base level) in case of 10 kg weight loss. Results of the aforementioned random parameters are based on normal distributions. The parameters *β*_4_ to *β*_6_ represent the two-way interactions of the *NONHEALTHGOAL* dummy (coded 1 for the non-health goal condition, and − 1 for the health goal condition) with respectively the variables *EXERCISE*, *DIET* _ *FLEX* and *WEIGHTLOSS* _ 5.

## Results

### Respondents

In total, 431 respondents (56%) who started the survey met the age (> = 18) and self-reported BMI (> = 25) requirements. From these respondents, 10 respondents (2.3%) completed the survey unusually fast (i.e., 2.5 min or less), 13 respondents (3.0%) reported an unusual height (i.e., shorter than 1.03 m), and one respondent did both (0.2%). We deleted the data of these 24 respondents, because we considered their responses as incorrect. Of the remaining 407 respondents, six respondents always chose program A (1.47%) and three always program B (0.74%) in all 16 choice tasks. We kept the data of these respondents, because we could not rule out that these choices reflected their true preferences. Analysis of respondents’ individual posterior estimates allowed us to determine a conservative upper bound of possible attribute non-attendance (we selected posteriors of values between − 0.05 and 0.05 as a proxy). We found that no respondent had posterior estimates in this range for two attributes at once (0.0%), while only few respondents had a posterior for a single attribute in this range (6.6%).

Respondents were randomly assigned to one of the two survey versions. We obtained data of 212 completes for the non-health goal condition (52%), and 195 completes for the health goal condition (48%). According to Bridges et al. [38], this is an acceptable number of respondents per subgroup. The weight of respondents ranged from 49 to 229 kg for the non-health goal condition, and from 58 to 180 kg for the health goal condition. Furthermore, respondents’ height ranged from 142 to 203 cm for the non-health goal condition, and from 153 to 203 cm for the health goal condition. Moreover, the average age of the respondents was respectively 49 and 48 years and ranged from 18 to 86 in the non-health goal condition and from 18 to 80 in the health goal condition. More information about the sample characteristics for each goal condition can be found in Table 3. Independent-samples t-tests and Chi-squared tests revealed that these sample characteristics did not significantly differ between the two goal conditions.

**Table 3:** Characteristics of the sample for the non-health goal and health goal conditions

Characteristics	Non-health goal condition			Health goal condition		
	N	(%)	Avg.	N	(%)	Avg.
Gender						
Male	99	(46.7)	–	100	(51.3)	–
Female	113	(53.3)	–	95	(48.7)	–
Education^a^						
Below bachelor	151	(71.2)	–	136	(69.7)	–
Bachelor or higher	60	(28.3)	–	58	(29.7)	–
Don’t know	1	(0.5)	–	1	(0.5)	–
Age (average in years)	–	–	49	–	–	48
Self-reported health (0–100 scale)	–	–	72	–	–	70
Reported importance of goals (1–5 scale)						
Losing weight	–	–	3.67	–	–	3.70
Feeling fitter	–	–	4.08	–	–	4.03
Reducing risk of overweight related diseases	–	–	4.01	–	–	4.03
Looking better	–	–	3.67	–	–	3.71
Increasing life expectancy	–	–	3.95	–	–	4.04
Improving endurance	–	–	4.07	–	–	4.09
Following a diet at the moment						
Yes	40	(18.9)	–	36	(18.5)	–
No	172	(81.1)	–	159	(81.5)	–
Reported physical activity in a week (in hours)^a^						
0–2	94	(44.3)	–	89	(45.6)	–
2–4	62	(29.2)	–	60	(30.8)	–
4–8	47	(22.2)	–	35	(18.0)	–
> 8	9	(4.2)	–	11	(5.6)	–
Ability to complete 6-month lifestyle program						
Yes	116	(54.7)	–	123	(63.1)	–
No	28	(13.2)	–	26	(13.3)	–
Don’t know	68	(32.1)	–	46	(23.6)	–
Participate in lifestyle program next 5 years						
Yes	47	(22.2)	–	51	(26.2)	–
No	60	(28.3)	–	50	(25.6)	–
Don’t know	105	(49.5)	–	94	(48.2)	–

^a^ Categories are combined

### DCE estimates

Based on the estimation results (see Table 4) we can determine if including goals in a DCE affects individuals’ choices via a moderating effect on other non-goal attributes. For the RPL model, we find significant effects for the variables *EXERCISE* and *WEIGHTLOSS* _ 5, with signs as expected across the two goal conditions. In particular, the parameter estimate for *EXERCISE* has a negative sign indicating that overweight individuals generally prefer to spend less hours per week on exercising.^2^ This is in line with our expectations, given that overweight individuals seem less willing to participate in physical activity than non-overweight individuals [39, 40]. Furthermore, as expected, the parameter of the *WEIGHTLOSS* _ 5 dummy has a negative sign, indicating that overweight individuals prefer to lose more (i.e., 10 kg) instead of less weight (i.e., 5 kg). The base effect of *DIET* _ *FLEX* is not significant in the RPL model across the two goal conditions. The significant individual-specific error terms (ν) of the random parameters for *EXERCISE*, *DIET* _ *FLEX*, and *WEIGHTLOSS* _ 5 indicate that individuals’ preferences for these attributes are heterogeneous.

**Table 4:** Results of the RPL model on lifestyle program choices

Variables	RPL model			
	Coefficients		Heterogeneity components	
	b	se	v	se
Constant	.21**	.05	–	–
Exercise	−.56**	.06	.90**	.06
Diet_Flex	.13	.18	2.87**	.19
Diet_Restricted (base)	–	–	–	–
Weightloss_5	−1.90**	.18	2.86**	.18
Weightloss_10 (base)	–	–	–	–
Nonhealthgoal_Exercise	.04	.05	–	–
Nonhealthgoal_Diet	.42*	.18	–	–
Nonhealthgoal_Weightloss	.03	.17	–	–
Observations	407			
LL	− 2621			
DF	10			
R^2^	.42			

RPL model indicates random parameter logit model

LL indicates log likelihood

DF indicates degrees of freedom

* Significant at *P* < 0.05. ** Significant at *P* < 0.01

The interaction effects (i.e., *NONHEALTHGOAL* _ *EXERCISE*, *NONHEALTHGOAL* _ *DIET*, and *NONHEALTHGOAL* _ *WEIGHTLOSS*) are important for our purposes, because they are directly relevant for answering the main question of this study. Interestingly, we find a positive significant effect (5% significance level), for the interaction *NONHEALTHGOAL* _ *DIET*, and non-significant effects for the other two interactions (i.e., *NONHEALTHGOAL* _ *EXERCISE* and *NONHEALTHGOAL* _ *WEIGHTLOSS*). This is evidence for our proposition that the type of goal can moderate the effect of non-goal attributes on individuals’ lifestyle program choices. In our results, the type of goal moderates the effect that the non-goal attribute diet has on lifestyle program choice. This significant interaction effect means that following a flexible diet (instead of a restricted diet) plays a more important role in individuals’ lifestyle program choices for individuals who were presented a non-health goal (i.e., looking better) than for individuals who were presented a health goal (i.e., increasing life expectancy).

## Discussion

The present study demonstrates how goals can be included in DCEs in the context of lifestyle program choices. Interestingly, we found a significant moderating effect of goals on the relative importance of non-goal attributes – specifically for the interaction of goal (non-health vs. health goal) with the diet attribute. An explanation for this effect may be that individuals who have as a goal to ‘look better by losing weight’, are less willing to follow a strict diet than individuals who have as a goal to ‘increase life expectancy by losing weight’. An explanation for the finding that the other interaction effects are not significant may be that individuals perceive these attributes as equally attractive means of pursuing their non-health versus health goals. Though little work exists on goals and DCEs, one exception is Swait, Franceschinis, and Thiene [41] in the environmental behavior literature. They estimated the effect of self-reported goals on non-goal attributes in a DCE about recreational site choices and found that spatial effects interacted with self-reported goal pursuit. However, they did not experimentally manipulate goals as we did in our study.

Some DCE-based studies included a goal-attribute to estimate main (instead of interaction) effects of goals on individuals’ lifestyle program choices. For example, Johnson et al. [14] used an attribute called ‘weight loss goal in a year’ and found a strong preference for programs with weight loss goals compared to programs with no weight loss goals. Furthermore, Ryan et al. [22] found that the attribute ‘short-term goal’ – with the attribute levels ‘look better’, ‘feel better’, and ‘look better and feel better’ – was not significant. The explanation they gave was that respondents may decide themselves about whether a given amount of weight loss would make them look and feel better. Consequently, they proposed valuing such ‘internal states’ indirectly as an alternative method [22]. Despite that a direct comparison with our study is difficult to make, their results contrast with our finding that goals do play a role in individuals’ lifestyle program choices, be it indirectly via non-goal attributes – here the diet attribute. Given that theoretically, goals are not a property of a lifestyle program (only program features are), but rather an ambition level defined by the individual, we advocate that it is better to incorporate goals as an interaction with program features rather than program main effects (as was done in earlier research).

We also compare the effects of the attributes diet, exercise, and weight loss with results from related literature. In contrast with Johnson et al. [14] and Chen et al. [23], who found a preference for a flexible instead of a restricted diet, we have not found a significant base effect of the diet attribute in our study. It can be that, independent of the goal (health or non-health), individuals do not have a specific base preference for diet type given a certain weight loss outcome. Furthermore, as in Johnson et al. [14] and Chen et al. [23], we find that exercising less is preferred over exercising more. This contrasts with the study of Alayli-Goebbels et al. [18] on health promotion programs (in which both health and non-health outcomes were included), because time investment for lifestyle change was not significant in that study. The explanation the authors offered for this finding was that time investment for lifestyle change may only be relevant for specific classes of respondents, but not on average for the entire sample [18]. Finally, as in Johnson et al. [14], Veldwijk et al. [19], and Wanders et al. [21], we find that losing more weight is preferred over losing less weight.

There are some limitations of this study. First, we found that following a flexible diet is more important for individuals who were presented with a non-health goal compared to individuals who were presented with a health goal. Our explanation for this finding is that individuals who had ‘looking better’ as a goal are less willing to follow a strict diet than individuals who had ‘increasing life expectancy’ as a goal. Despite that this explanation may be in line with the finding of Laran, Janiszewski, and Salerno [25] that conscious goal pursuit increases the likelihood of choosing alternatives that are most consistent with the goal, we do not have data to prove this reasoning. The aforementioned explanation seems plausible given that individuals were presented one specific goal in all choice tasks, which most likely made them highly conscious of that goal. However, it would be interesting in future research to study the reasoning behind individuals’ choices. For this purpose, a think aloud approach could be used to obtain qualitative statements that help understanding individuals’ choice processes [42].

Second, we used relatively easy choice tasks in which the attribute levels of only three attributes varied in the design and one goal was presented to respondents. This design reflected the main research question in this study. For a more extensive analysis of lifestyle program choice behavior, however, it would be interesting to extend the design of the DCE by including more non-goal attributes and/or multiple goals. The inclusion of multiple goals can reflect the fact that individuals may have in mind multiple different benefits from participating in a specific lifestyle program.

Third, attributes and attribute levels were selected based on their successful use in the DCE literature on individuals’ preferences for lifestyle programs, and more specifically in the Dutch context in which we investigated individuals’ decisions. However, the use of focus groups or other qualitative research for testing and selecting the attributes and attribute levels could have been valuable to further inform and validate this aspect of our study.

Fourth, respondents who had a different real-life goal than the goal they were externally assigned to may have had more difficulties completing the choice tasks. However, the goals were, on average, all considered important by respondents. Furthermore, respondents were presented the same goal in all choice tasks, which made it easier for them to make choices with this specific goal in mind. If respondents would have been presented different goals over the choice tasks, or different goals within choice tasks, this could have made the choice tasks too unrealistic. Therefore, in this paper, we specifically propose an approach that uses a fixed goal in all choice tasks and only let goals vary between the experimental conditions. Future research could include an external validity check of the proposed DCE-based approach.

Fifth, respondents were asked to make explicit choices between the two lifestyle programs to obtain balanced and complete responses to all choice sets. This approach allowed us to obtain full choice observations in each goal condition, which enhanced comparability. An alternative approach, that emphasizes lifestyle program demand estimation, would be to include an opt-out in each choice set to reflect that individuals may have the possibility of not participating in any specific lifestyle program [20].

Finally, in discrete choice experiments individuals are asked to make choices between hypothetical alternatives [38]. Despite that stated preferences obtained from a DCE can adequately predict actual behavior in a public health context [24], individuals' DCE-based choices may not always reflect their real-life decisions. Therefore, it is important to further establish the external validity of the findings in real-world decisions. For example, future research could sample individuals who already participate in lifestyle programs, establish what goals these individuals hold for lifestyle program participation, and what program choices they have made when they selected their current program. These revealed choices and goals could then be used to validate responses in a separate sample related to DCE program choices that incorporates goals according to the method proposed in this paper.

## Conclusions

This study proposes an approach of how to include goals in DCEs and applies it in the context of individuals’ lifestyle program choices. As anticipated, the results of our DCE-based study show that introducing different goals (i.e., non-health vs. health goals) affects individuals’ choices via a moderating effect on non-goal attributes. Interestingly, we find that a flexible diet is more important for individuals who were presented a non-health goal (looking better) than for individuals who were presented a health goal (increasing life expectancy). The finding that incorporating goals in a DCE may affect individuals’ choices via an effect on non-goal attributes offers interesting insights for both researchers that plan to set-up DCEs and decision makers that use DCE-based results. The significant interaction of goal with the diet attribute implies that, depending on their goal, individuals may attach different weights to the same attribute.

In practice, policy makers can use information on such goal interactions for developing new policies and communication strategies to target population segments that have different goals. For this purpose, we propose the following practical approach. First, policy makers could determine which goals are perceived as important in the population of interest based on a representative sample. Second, the relative importance of each goal could be examined. Third, several population segments could be defined based on who ranked a goal as most important (see step 2). Finally, these population segments could be targeted differently in communication and policy options. Different communication strategies and other policy options could be used for each goal-based segment.

In the present study, goal-setting was experimentally manipulated. Further research that tries to unify goal-setting and discrete choice could focus on developing this DCE approach while also estimating econometric models to predict individuals’ goal-setting behavior. It would also be interesting to investigate if, and if so how, the inclusion of multiple goals (instead of one specific goal) affects individuals’ choices. Finally, we hope that this current research can encourage future research to study the impact of goals in DCEs in other health contexts than lifestyle programs.

## Supplementary information

**Additional file 1.** Survey instrument.

## Data Availability

The dataset used for the current study is available from the corresponding author on reasonable request.

## Abbreviations

BMI: Body mass index
DCE: Discrete choice experiment
MNL model: Multinomial logit model
RPL model: Random parameter logit model

## Footnotes

1 In the survey, we also collected data on a condition without a goal. This data was not used in this paper, since our focus is on the proposed method of including goals in DCEs and how different goals impact individuals’ preferences.

2 We also tested a model in which dummy coding was used for the variable exercise. However, this did not improve model fit.
