# Supplementary material for: The impact of health vs. non-health goals on individuals’ lifestyle program choices: a discrete choice experiment approach
Source: BMC Public Health. 2020 Mar 30;20:411. doi: 10.1186/s12889-020-8416-3 (PMC7106806; doi:10.1186/s12889-020-8416-3)
Supplement: Supplementary file 1 — Additional file 1. Survey instrument. [file 12889_2020_8416_MOESM1_ESM.pdf]

Survey instrument (translated from Dutch)

Introduction

This survey is part of a scientific study conducted by researchers of the Erasmus University Rotterdam. Participating in this survey is completely voluntary. Therefore, you can end the survey at any time.

Purpose

The purpose of this survey is to determine individuals' preferences regarding lifestyle programs.

Use of PC or laptop

To be sure that the survey runs smoothly it is important that you use a PC or laptop. Please note that the survey cannot be completed on your mobile phone.

What kind of questions can you expect?

First, you will be asked about your gender, age, height and weight, so that it can be determined if you belong to the target group. If so, you will get a brief explanation of the different characteristics of the lifestyle programs that appear in the survey. Consequently, we will ask you to complete an example choice task and 16 main choice tasks. In all these choice tasks, you need to choose between two lifestyle programs that differ on several characteristics. Furthermore, at the end of the survey, several general questions will be asked.

Confidentiality and anonymity

Note that you will not be asked for information that allows the researchers to identify you personally. Furthermore, your answers will always be processed anonymously and only be used for analysis on an aggregate level.

Do you agree?

Please click on "I agree" if you have understood and agree with the aforementioned information. Then, the survey will continue. Please click on “I do not agree” if you do not agree with this information. In this case, the survey will be ended.

□ I agree

□ I do not agree

The average weight of people in the Netherlands has increased in recent years. At this moment, about 50% of the Dutch adults are overweight, and about 14% of the Dutch adults are obese (heavy overweight).

This questionnaire is meant for adults with overweight or heavy overweight. To determine if you belong to the target group, we first ask you several questions.

What is your age?

… years [18-100]

What is your gender?

□ Male

□ Female

What is your weight?

… kg [0-250]

What is your height?

… cm [0-250]

Based on your previous answers, your BMI is calculated and presented below:

Your BMI is …

Is your BMI (number above) higher (or equal to) or lower than 25?

□ My BMI is higher than or equal to 25

□ My BMI is lower than 25

Overweight people can be helped with losing weight by participating in a lifestyle program. In this survey, we are interested in your preferences with respect to different lifestyle programs. The features of these programs and their corresponding levels will be explained first. After this explanation, we ask you to complete several choice tasks in which you have to choose between two different lifestyle programs in each choice task. After these choice tasks, several general questions will follow at the end of the survey.

Diet

Following a diet is one of the features of a lifestyle program. In the lifestyle programs of this survey, we distinguish between a 'flexible low-calorie diet' and a 'restricted diet'. A flexible low-calorie diet means that you are flexible in what you eat, as long as you do not take more than the maximum number of calories. In a restricted diet, you strictly follow the dietitian's suggestions (this means that you are not flexible in what you eat).

Exercise per week

Exercising (physical activity) is another feature of a lifestyle program. In the lifestyle programs of this survey we distinguish between ‘2 hours of exercise’, ‘4 hours of exercise’, and ‘6 hours of exercise’ per week.

Expected weight loss

The weight you lose also is a feature of a lifestyle program. In the lifestyle programs of this survey, we distinguish between ‘5kg’ and ’10kg’ weight loss. This weight loss represents the total weight you will probably lose by participating in the lifestyle program.

(The text below was presented in the non-health goal condition)

Goal

The goal is another feature of a lifestyle program. The goal in the lifestyle programs of this survey is ‘looking better by losing weight’.

(The text below was presented in the health goal condition)

Goal

The goal is another feature of a lifestyle program. The goal in the lifestyle programs of this survey is ‘increasing life expectancy by losing weight’.

We now continue with the choice tasks.

Please always imagine the following when you complete the choice tasks:

You would like to lose weight, but do not know where to begin. Therefore, you have a conversation with your general practitioner. During this conversation, your general practitioner explains that you could participate in a lifestyle program. The type of program that your general practitioner recommends has the following characteristics:

The program lasts for six months.

The total costs of the lifestyle program will be fully reimbursed (basic health insurance).

During the program, you have six counseling sessions (once per month) with your lifestyle coach.

During these sessions, your progress will be discussed and you will receive useful tips.

The lifestyle program contains an exercise activity that you would like to participate in.

Both individual and group exercises are possible.

The exercise activities take place during a time and location that suits you.

You are highly interested in participating in this type of lifestyle program and therefore started searching for the program of your preference. In this process, you come across programs that only differ based on the previously discussed features (i.e., diet, exercise per week, and expected weight loss).

On the next pages, we show you two different lifestyle programs and ask you to choose between these two programs.

We start with an example choice task. In the example choice task below you have to make a choice between programs A and B by selecting your preferred program.

(The example choice task below was presented in the non-health goal condition)

Example choice task. Which lifestyle program do you prefer: program A or B?

**Features**	**Program A**	**Program B**
Diet	restricted diet	flexible low-calorie diet
Exercise per week	2 hours of exercise	6 hours of exercise
Expected weight loss	10 kg	10 kg
Goal	looking better by losing weight	looking better by losing weight
I prefer:	Program A<br>□	Program B<br>□

Keep your cursor ***here*** *to read the description of the situation again.*

Keep your cursor ***here*** *to read the information about the features of the lifestyle programs again.*

(The example choice task below was presented in the health goal condition)

Example choice task. Which lifestyle program do you prefer: program A or B?

**Features**	**Program A**	**Program B**
Diet	restricted diet	flexible low-calorie diet
Exercise per week	2 hours of exercise	6 hours of exercise
Expected weight loss	10 kg	10 kg
Goal	increasing life expectancy by losing weight	increasing life expectancy by losing weight
I prefer:	Program A<br>□	Program B<br>□

Keep your cursor ***here*** *to read the description of the situation again.*

Keep your cursor ***here*** *to read the information about the features of the lifestyle programs again.*

We now continue with the real choice tasks.

Please always imagine the following when you complete the choice tasks:

You would like to lose weight, but do not know where to begin. Therefore, you have a conversation with your general practitioner. During this conversation, your general practitioner explains that you could participate in a lifestyle program. The type of program that your general practitioner recommends has the following characteristics:

The program lasts for 6 months.

The total costs of the lifestyle program will be fully reimbursed (basic health insurance).

During the program, you have 6 counseling sessions (once per month) with your lifestyle coach. During these sessions your progress will be discussed and you will receive useful tips.

The lifestyle program contains an exercise activity that you would like to participate in.

Both individual and group exercises are possible.

The exercise activities take place during a time and location that suits you.

You are highly interested in participating in this type of lifestyle program and therefore started searching for the program of your preference. In this process, you come across programs that only differ based on the previously discussed features (i.e., diet, exercise per week, and expected weight loss).

In the next 16 choice tasks, you have to make a choice between the programs A and B by selecting your preferred program (just like the example choice task).

(We do not show the specific attribute levels for the non-goal attributes here, because they were the same for the two goal conditions)

(The choice tasks below were presented in the non-health goal condition)

Choice task 1-16. Which lifestyle program do you prefer: program A or B?

**Features**	**Program A**	**Program B**
Diet		
Exercise per week		
Expected weight loss		
Goal	looking better by losing weight	looking better by losing weight
I prefer:	Program A<br>□	Program B<br>□

Keep your cursor ***here*** *to read the description of the situation again.*

Keep your cursor ***here*** *to read the information about the features of the lifestyle programs again.*

(The choice tasks below were presented in the health goal condition)

Choice task 1-16. Which lifestyle program do you prefer: program A or B?

**Features**	**Program A**	**Program B**
Diet		
Exercise per week		
Expected weight loss		
Goal	increasing life expectancy by losing weight	increasing life expectancy by losing weight
I prefer:	Program A ☐	Program B ☐

*Keep your cursor **here** to read the description of the situation again.*

*Keep your cursor **here** to read the information about the features of the lifestyle programs again.*

You have completed all choice tasks. Now, several general questions follow.

How important are the following goals for you? (1 = very unimportant, 5 = very important)

- Losing weight
- Feeling fitter
- Reducing risk of overweight related diseases
- Looking better
- Increasing life expectancy
- Improving endurance

How healthy do you normally feel? (0 = worst health state, 100 = best health state)

What is the highest education level you have obtained?

□ Primary education

□ Primary / preparatory vocational education

□ General secondary education

□ Secondary vocational education

□ Higher general secondary education

□ Pre-university education

□ Higher professional education

□ Scientific education

□ Don't know

Do you currently follow a diet?

□ Yes

□ No

How many hours do you (on average) spend on physical activity (exercising) per week?

□ 0-2 hours

□ 2-4 hours

□ 4-6 hours

□ 6-8 hours

□ More than 8 hours

Do you think you can complete a lifestyle program that lasts 6 months?

□ Yes

□ No

□ Don’t know

Do you plan to participate in a lifestyle program in the next 5 years?

□ Yes

□ No

□ Don’t know

You almost completed the questionnaire. Please select ‘save my answers’ to save your answers, and only then continue to the next page.

□ Save my answers.

Thank you for your participation!
